# Meat consumption and risk of primary hip and knee joint replacement due to osteoarthritis: a prospective cohort study

**DOI:** 10.1186/1471-2474-12-17

**Published:** 2011-01-16

**Authors:** Yuanyuan Wang, Julie Anne Simpson, Anita E Wluka, Dallas R English, Graham G Giles, Stephen Graves, Flavia M Cicuttini

**Affiliations:** 1 Department of Epidemiology and Preventive Medicine, School of Public Health and Preventive Medicine, Monash University, Alfred Hospital, Melbourne, VIC 3004, Australia; 2 Centre for Molecular, Environmental, Genetic and Analytic Epidemiology, School of Population Health, University of Melbourne, Carlton, VIC 3053, Australia; 3 Cancer Epidemiology Centre, The Cancer Council Victoria, Carlton, VIC 3053, Australia; 4 Department of Orthopaedic Surgery, University of Melbourne, Royal Melbourne Hospital, Melbourne, Australia; 5 AOA National Joint Replacement Registry; Discipline of Public Health, School of Population Health & Clinical Practice, University of Adelaide, SA 5005, Australia

## Abstract

**Background:**

There is emerging evidence for a beneficial effect of meat consumption on the musculoskeletal system. However, whether it affects the risk of knee and hip osteoarthritis is unknown. We performed a prospective cohort study to examine the relationship between meat consumption and risk of primary hip and knee replacement for osteoarthritis.

**Methods:**

Eligible 35,331 participants were selected from the Melbourne Collaborative Cohort Study recruited during 1990-1994. Consumption of fresh red meat, processed meat, chicken, and fish was assessed using a food frequency questionnaire. Primary hip and knee replacement for osteoarthritis during 2001-2005 was determined by linking the cohort records to the Australian National Joint Replacement Registry.

**Results:**

There was a negative dose-response relationship between fresh red meat consumption and the risk of hip replacement (hazard ratio (HR) 0.94 per increase in intake of one time/week, 95% confidence interval (CI) 0.89-0.98). In contrast, there was no association with knee replacement risk (HR 0.98, 95% CI 0.94-1.02). Consumption of processed meat, chicken and fish were not associated with risk of hip or knee replacement.

**Conclusion:**

A high level consumption of fresh red meat was associated with a decreased risk of hip, but not knee, joint replacement for osteoarthritis. One possible mechanism to explain these differential associations may be via an effect of meat intake on bone strength and hip shape. Further confirmatory studies are warranted.

## Background

Whilst the consumption of red meat is often recommended for its iron content [1], there is emerging evidence that red meat and processed meat are associated with carcinogenesis at several anatomic sites and increased mortality [2-5]. The mechanisms for these adverse effects are various but have been related to the constituent nutrients of red meat and methods of processing and cooking. Meat is a source of several multisite carcinogens including N-nitroso compounds, heterocyclic amines, and polycyclic aromatic hydrocarbons, some of which are formed during high-temperature cooking of meat [2]. Iron in red meat may increase oxidative damage and increase the formation of N-nitroso compounds [6]. Meat is also a major source of saturated fat which has been positively associated with cancer [7]. Current dietary recommendations are to consume small to moderate amounts of red meat and processed meat as a way of reducing the risk of a number of chronic diseases including cardiovascular disease and cancer [8].

Meat consumption may have beneficial effects on the musculoskeletal system since there is evidence that high animal protein is associated with high bone mineral density and low risk of hip fracture [9-11]. Osteoarthritis (OA) is a common chronic joint disease involving the whole joint including the articular cartilage, subchondral bone and soft tissues. There is evidence that dietary nutrients may protect against OA [12]. However, it is unknown whether meat consumption may affect the risk of OA, and the existing evidence is conflicting. A recent cross-sectional study reported a higher prevalence of degenerative arthritis associated with greater meat consumption [13], whereas another cross-sectional study found no relationship between consumption of meat/poultry and fish and the prevalence of knee OA [14].

Total joint replacement is the only established treatment for those with symptomatic end-stage OA where conservative management fails, thus it may be considered a proxy outcome measure for severe end-stage OA. We analyzed data from the Melbourne Collaborative Cohort Study (MCCS) on consumption of red meat, processed meat, chicken, and fish to determine the relation to the risk of primary knee and hip joint replacement due to OA.

## Methods

### The cohort

The MCCS is a prospective cohort study of 41,528 people (17,049 men) aged between 27 and 75 years at baseline, 99.3% of whom were aged 40 - 69 years [15]. Participants were recruited via Electoral Rolls (registration to vote is compulsory for Australian adults), advertisements, and community announcements in the local media (e.g., television, radio, newspapers), between 1990 and 1994. Southern European migrants to Australia (including 5,425 from Italy and 4,535 from Greece) were deliberately over-sampled to extend the range of lifestyle exposures and to increase genetic variation. The study protocol was approved by The Cancer Council Victoria's Human Research Ethics Committee. All participants gave written consent to participate and for the investigators to obtain access to their medical records.

Follow-up was conducted by record linkage to Electoral Rolls, electronic phone books and the Victorian Cancer Registry and death records. To update lifestyle exposures, the cohort was followed up by mailed questionnaire and where necessary by telephone from 1995 to 1998 (first follow-up) and by face-to-face interviews from 2003 to 2007 (second follow-up). From 2003 onwards, 28,046 study participants (68% of the original MCCS participants) took part in the second follow-up.

### Dietary assessment

At baseline, dietary intake over the previous 12 months was estimated using a 121-item food frequency questionnaire (FFQ) specifically developed for the MCCS. The FFQ was developed from a study of weighed food records in a sample of 810 Melbourne residents of similar age and ethnic origin to the MCCS cohort [16]. The FFQ included 22 items on intake of fresh red meat, processed meat, chicken, and fish. Fresh red meat was defined as veal or beef schnitzel, roast beef or veal, beef steak, rissoles (meat balls) or meatloaf, mixed dishes with beef, roast lamb or lamb chops, mixed dishes with lamb, roast pork or pork chops, and rabbit or other game (rarely consumed). Processed meat included salami or continental sausages, sausages or frankfurters, bacon, ham including prosciutto, corned beef, and manufactured luncheon meats. Chicken included roast or fried chicken, boiled or steamed chicken, and mixed dishes with chicken. Fish included steamed, grilled, or baked fish, fried fish, smoked fish, and canned fish including tuna, salmon, and sardines. At the MCCS first follow up, basic questions (not complete FFQ as used at MCCS baseline) about the frequency of meat consumption (fresh red meat, chicken, and fish) over the last year were asked.

Nutrient intakes were calculated using standard sex-specific portion sizes from the weighed food records [16]. The energy and nutrient contents in food were computed using Australian food composition tables [17]. Energy from alcoholic beverages was added to that calculated from the FFQ. Fatty acid composition of foods was obtained from the Royal Melbourne Institute of Technology fatty acid database [18]. Carotenoid data were obtained from the 1998 United States Department of Agriculture database [19].

To estimate the reproducibility of the FFQ, between July 1992 and June 1993, 275 participants were invited to participate in a study that required completing a second FFQ 12 months after baseline. Of these, 242 (88%) completed the second FFQ. The weighted kappa statistics for the reproducibility of the quartiles of meat intake were 0.42 (95% CI, 0.30-0.55) for fresh red meat, 0.60 (0.48-0.73) for processed meat, 0.44 (0.32-0.56) for chicken, and 0.48 (0.35-0.61) for fish.

### Assessment of demographic, lifestyle and anthropometric factors

At baseline, a structured interview schedule was used to obtain demographic and lifestyle information including date of birth, country of birth, smoking, alcohol consumption, current physical activity during leisure time, and education. Height and weight were measured once at baseline attendance for each participant according to written protocols based on standard procedures [20]. Weight was measured to the nearest 0.1 kg using digital electronic scales, height was measured to the nearest 1 mm using a stadiometer. Body mass index (BMI) was calculated as weight in kilograms divided by the square of height in meters.

At the MCCS second follow-up, the participants were asked questions enquiring about their first joint replacement surgery: Have you ever had a hip replacement? When did you have your first hip replacement? Have you ever had a knee replacement? When did you have your first knee replacement?

### Study participants

Of the 41,528 participants recruited, 6197 (14.9%) were excluded from analysis because they: reported extreme energy intake (<1^st ^percentile or >99^th ^percentile); reported an acute myocardial infarct, angina or diabetes at baseline and were likely to have changed their diet; had missing dietary data; died or left Australia prior to January 1, 2001; at the MCCS second follow-up had reported a primary joint replacement prior to January 1, 2001; had left Australia before the date of having a primary joint replacement; or had the first recorded procedure being a revision joint replacement as recorded in the Australian Orthopaedic Association National Joint Replacement Registry (AOA NJRR), thus leaving 35,331 participants available for analysis.

### Identification of incident primary knee and hip joint replacement

Cases were identified from the AOA NJRR. The implementation of the AOA NJRR commenced in 1999 and was introduced in a staged state by state approach which was completed nationally by mid 2002. Victorian data collection commenced in 2001. The Registry monitors the performance and outcome of both hip and knee replacement surgery in Australia. It has detailed information on the prostheses and surgical technique used and the clinical situation that it was used in for both primary and revision joint replacement [21]. By using detailed matching technology it is able to determine the success or otherwise of the joint replacement surgery. Although data collection for the Registry is voluntary, it receives cooperation from all hospitals undertaking joint replacement surgery [21].

The AOA NJRR validates its data by using both internal systems and external data sources. The most important external data source is state health department data. Validation of registry data against health department recorded data involves a sequential multilevel matching process. Following the validation process and the retrieval of unreported records, the Registry collects the most complete set of data relating to hip and knee replacement in Australia [21].

Identifying information of MCCS participants, including first name, last name, date of birth, and gender, was provided to the staff at the AOA NJRR in order to identify those MCCS participants who had had a primary or revision joint replacement between January 1, 2001 which is when the Registry commenced Victorian data collection, and December 31, 2005. The matching was performed on these data provided using U.S. Bureau of the Census Record Linkage Software. Exact matches were identified and probabilistic matches were reviewed. The staff from the AOA NJRR forwarded this information to MCCS and it was then added to the MCCS database.

The study was approved by The Cancer Council Victoria's Human Research Ethics Committee and the Standing Committee on Ethics in Research Involving Humans of Monash University.

### Statistical analysis

Cox proportional hazards regression models were used to estimate the hazard ratios (HR) for first recorded primary joint replacement associated with individual meat consumption after adjustment for confounding variables. Follow-up for primary joint replacement (i.e. calculation of person-time) began at January 1, 2001, and ended at date of first primary joint replacement for OA or date of censoring. Participants were censored at either the date of first primary joint replacement performed for indications other than OA, the date of death, the date left Australia, or end of follow-up (i.e. December 31, 2005 (the date that ascertainment of joint replacement by NJRR was complete), whichever came first.

All meat consumption variables were analysed categorically, based on approximate quartiles of the distribution of weekly frequency of consumption, with the first quartile used as the reference category. Linear associations between meat consumption and the risk of joint replacement were investigated by comparing the regression models with meat consumption as a categorical variable and a pseudo-continuous variable using the likelihood ratio test. Tests for trend across categories of meat consumption were calculated using meat consumption as a pseudo-continuous variable, assuming that, within each quarter all participants consumed at its median frequency. We also calculated the ratio of frequency of consumption of fresh red meat to the combined frequency of consumption of chicken and fish and divided it into groups based on quartiles. To estimate HR separately for knee and hip replacement risk and to test for heterogeneity, Cox models based on competing risks were fitted using a data duplication method [22].

Age, gender, BMI, country of birth, and energy intake (kj/d) were included in all models. Other potential confounding variables were included in all the definitive analyses if they changed the HR of any of the meat consumption variables for either hip or knee joint replacement risk by at least 5%. First, education, current level of physical activity, smoking (current/past/never), and alcohol consumption (g/d) were added. The HR changed < 5%, thus, none of these variables were retained for further analysis. Second, multi-vitamins and fish oil supplement were added. HR changed < 5%. Third, fruit, vegetable, vitamin C, vitamin E, beta-carotene, and polyunsaturated fatty acids were added to the model, one at a time. HR changed < 5%. Thus none of the dietary variables were retained for further analysis.

To test whether associations between meat consumption and the risk of joint replacement were modified by country of birth, gender or educational level, interactions between country of birth, gender or educational level and meat consumption were fitted, and tested using the likelihood ratio test. Tests based on Schoenfeld residuals and graphical methods using Kaplan-Meier curves showed no evidence that proportional hazard assumptions were violated for any analysis. All statistical analyses were performed using Stata (Intercooled Stata 9.2 for Windows, StataCorp LP., College Station, TX, USA).

## Results

**Figure 1:**
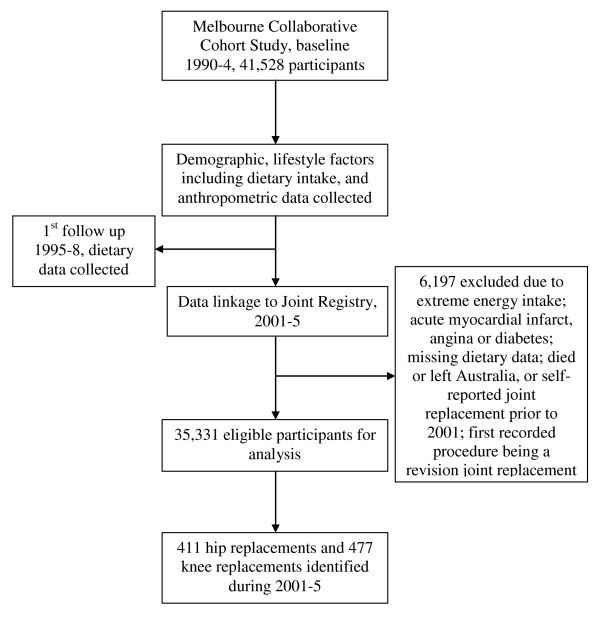
**Study profile**.

The study profile is shown in Figure 1. Over an average of 4.8 (SD 0.7) years of follow-up per person (2001-5), 888 participants were identified with incident primary joint replacement for OA including 411 hip replacements and 477 knee replacements. The baseline (1990-4, an average of 12.6 years prior to the joint replacement) demographic characteristics and meat consumptions of the study population are presented in Tables 1, 2 and 3. Meat was eaten more frequently by men than by women: men's weekly median frequencies of consumption were 5.0 times for fresh red meat and 2.5 times for processed meat compared to 4.0 and 2.0 times for women. Italian and Greek immigrants consumed fresh red meat (median 5.0 versus 4.5 times/week) and chicken (median 2.0 versus 1.5 times/week) more frequently than those born in Australia and United Kingdom. Participants with lower education level consumed fresh red meat more frequently than those with a higher level of education (median 4.5 versus 4.0 times/week). The meat consumptions at baseline and first follow up were moderately correlated, with the correlation coefficient ranging from 0.37 to 0.40 for fresh red meat, chicken and fish.

**Table 1:** Baseline demographic characteristics of study population

	Baseline population n = 35331	1st follow up population n = 29910
Age of entering MCCS	54.5 [8.6]	54.5 [8.6]
Age of entering joint replacement cohort	62.2 [8.8]	62.2 [8.8]
Women, number (%)	21580 (61.1)	18305 (61.2)
Body mass index, kg/m^2^	26.7 [4.4]	26.5 [4.3]
Country of birth, number (%)		
Australia/United Kingdom	27256 (77.1)	24124 (80.7)
Italy/Greece	8075 (22.9)	5786 (19.3)
Education, number (%)		
Primary and some secondary	19606 (55.8)	15869 (53.3)
Completed secondary and degree/diploma	15533 (44.2)	13921 (46.7)

Values are reported as mean [SD], or number (%).

MCCS: The Melbourne Collaborative Cohort Study

**Table 2:** Baseline meat consumption of study population

	Primary hip replacement n (%)*	Primary knee replacement n (%)*	Total number in each category
Fresh red meat intake			
<3.0 times/week	94 (1.2)	92 (1.2)	7576
3.0 - 4.4 times/week	123 (1.3)	135 (1.4)	9371
4.5 - 6.4 times/week	114 (1.2)	146 (1.5)	9661
≥6.5 times/week	80 (0.9)	104 (1.2)	8723
Processed meat intake			
<1.5 times/week	97 (1.4)	85 (1.2)	7154
1.5 - 1.9 times/week	84 (1.2)	96 (1.3)	7324
2.0 - 3.9 times/week	132 (1.1)	183 (1.5)	11879
≥4.0 times/week	98 (1.1)	113 (1.3)	8974
Chicken intake			
<1.5 times/week	118 (1.1)	152 (1.4)	10680
1.5 - 1.9 times/week	99 (1.5)	84 (1.3)	6713
2.0 - 3.4 times/week	92 (0.9)	132 (1.4)	9766
≥3.5 times/week	102 (1.3)	109 (1.3)	8172
Fish intake			
<1.0 times/week	74 (1.1)	97 (1.4)	6764
1.0 - 1.4 times/week	105 (1.3)	110 (1.3)	8229
1.5 - 2.4 times/week	133 (1.1)	166 (1.4)	11997
≥2.5 times/week	99 (1.2)	104 (1.3)	8341
Ratio of intake of fresh red meat to chicken and fish			
<0.77	127 (1.4)	125 (1.4)	8867
0.77 - 1.25	103 (1.1)	118 (1.3)	9336
1.26 - 2.00	100 (1.1)	126 (1.3)	9512
≥2.01	81 (1.1)	108 (1.4)	7616

*number (%) of participants with primary hip or knee replacement in each dietary meat category

**Table 3:** Baseline demographic characteristics by consumption of fresh red meat

	< 3.0 times/week n = 7576	3.0 - 4.4 times/week n = 9371	4.5 - 6.4 times/week n = 9661	≥6.5 times/week n = 8723
Age of entering MCCS	54.1 [8.8]	54.4 [8.6]	54.6 [8.5]	54.8 [8.4]
Age of entering joint replacement cohort	61.7 [9.0]	62.1 [8.8]	62.3 [8.7]	62.7 [8.6]
Women, number (%)	5196 (24.1)	6020 (27.9)	5791 (26.8)	4573 (21.2)
Body mass index, kg/m^2^	26.1 [4.4]	26.5 [4.3]	26.8 [4.3]	27.4 [4.4]
Country of birth, number (%)				
Australia/United Kingdom	6088 (22.3)	7463 (27.4)	7608 (27.9)	6097 (22.4)
Italy/Greece	1488 (18.4)	1908 (23.6)	2053 (25.4)	2626 (32.5)
Education, number (%)				
Primary and some secondary	3818 (19.5)	5053 (25.8)	5409 (27.6)	5326 (27.2)
Completed secondary and degree/diploma	3725 (24.0)	4262 (27.4)	4211 (27.1)	3335 (21.5)

Values are reported as mean [SD], or number (%).

MCCS: The Melbourne Collaborative Cohort Study

Table 4 shows the hazard ratios according to baseline meat consumption for hip and knee joint replacement separately. There was a dose-response relationship observed between fresh red meat consumption and the risk of hip joint replacement: the HR were 1.00, 0.88, and 0.69 in the 2^nd^, 3^rd ^and 4^th ^quartile groups respectively compared with the 1^st ^quartile group (P for trend 0.006), the highest quartile of fresh red meat consumption was significantly associated with a decreased risk of hip joint replacement compared with the bottom quartile [HR 0.69, 95% confidence interval (CI) 0.50 - 0.94]. No dose-response relationship was observed for the association between consumption of fresh red meat and the risk of knee joint replacement: HR were 1.13, 1.15, 0.91 in the 2^nd^, 3^rd ^and 4^th ^quartile groups compared with the bottom quartile group. Consumption of processed meat, chicken or fish had little or no association with the risk of either hip or knee replacement.

**Table 4:** Hazard ratios of hip and knee joint replacement by baseline meat consumption

	Quartile of frequency of consumption at baseline [Hazard ratio (95% CI)^a^]			Hazard ratio (95% CI) for increase in intake of one time per week^b^	P for trend	Homogeneity of trends^c^
	2	3	4 (highest)			
Fresh red meat						
Hip replacement	1.00 (0.77, 1.31)	0.88 (0.66, 1.15)	0.69 (0.50, 0.94)	0.94 (0.89, 0.98)	0.006	0.14
Knee replacement	1.13 (0.86, 1.47)	1.15 (0.88, 1.49)	0.91 (0.68, 1.22)	0.98 (0.94, 1.02)	0.33	
Processed meat						
Hip replacement	0.75 (0.56, 1.004)	0.71 (0.54, 0.93)	0.76 (0.57, 1.02)	0.96 (0.91, 1.02)	0.23	0.34
Knee replacement	0.98 (0.73, 1.31)	1.13 (0.87, 1.46)	1.00 (0.75, 1.33)	1.00 (0.95, 1.05)	1.00	
Chicken						
Hip replacement	1.46 (1.12, 1.92)	0.94 (0.71, 1.24)	1.22 (0.93, 1.60)	1.03 (0.96, 1.11)	0.39	0.59
Knee replacement	0.96 (0.74, 1.26)	1.04 (0.83, 1.32)	1.01 (0.79, 1.30)	1.01 (0.94, 1.08)	0.88	
Fish						
Hip replacement	1.17 (0.87, 1.57)	1.02 (0.76, 1.36)	1.06 (0.78, 1.44)	1.00 (0.91, 1.09)	0.97	0.43
Knee replacement	0.93 (0.71, 1.23)	0.97 (0.75, 1.25)	0.85 (0.64, 1.12)	0.95 (0.87, 1.03)	0.24	
Ratio of fresh red meat to chicken and fish						
Hip replacement	0.80 (0.62, 1.04)	0.77 (0.59, 0.996)	0.68 (0.51, 0.90)	0.87 (0.77, 0.97)	0.01	0.12
Knee replacement	0.93 (0.72, 1.20)	0.98 (0.76, 1.26)	0.92 (0.71, 1.19)	0.97 (0.88, 1.08)	0.62	

^a^Adjusted for age, gender, body mass index, country of birth, and energy intake using Cox's proportional hazard model. See Table 2 for ranges of each quartile.

^b^For ratio of consumption of fresh red meat to chicken and fish, hazard ratio is for a one-unit increase in the ratio.

^c^Test of homogeneity of trends for knee and hip joint replacement.

The ratio of fresh red meat consumption to chicken and fish consumption was also examined (Table 4). There was a negative dose-response association observed for the risk of hip replacement (HR of 0.87 for increase of 1 unit of the ratio, 95% CI 0.77 - 0.97, P for trend 0.01), but no association observed for the risk of knee replacement (HR 0.97, 95% CI 0.88 - 1.08, P for trend 0.62). The statistical evidence for heterogeneity in these dose-response associations between the hip and knee was weak (test for homogeneity of trends, P = 0.12).

Similar results were observed when examining the meat consumption at first follow up (Table 5). Although the same direction and magnitude of association between fresh red meat consumption and risk of hip replacement were observed (HR 0.94, 95% CI 0.87 - 1.01), this protective association was not statistically significant (P = 0.11).

**Table 5:** Hazard ratios of hip and knee joint replacement by first follow up meat consumption

	Quartile of frequency of consumption at first follow up [Hazard ratio (95% CI)^a^]			Hazard ratio (95% CI) for increase in intake of one time per week	P for trend	Homogeneity of trends^b^
	2	3	4 (highest)			
Fresh red meat						
Hip replacement	0.96 (0.72, 1.28)	0.78 (0.58, 1.04)	0.81 (0.59, 1.10)	0.94 (0.87, 1.01)	0.11	0.02
Knee replacement	0.82 (0.61, 1.12)	1.02 (0.78, 1.35)	1.23 (0.93, 1.63)	1.07 (0.99, 1.14)	0.07	
Chicken						
Hip replacement	1.11 (0.84, 1.48)	0.97 (0.72, 1.31)	1.06 (0.73, 1.53)	0.99 (0.89, 1.10)	0.84	0.64
Knee replacement	1.02 (0.78, 1.34)	1.00 (0.75, 1.32)	1.11 (0.78, 1.57)	1.03 (0.92, 1.14)	0.64	
Fish						
Hip replacement	0.90 (0.70, 1.16)	1.19 (0.90, 1.58)	1.33 (0.93, 1.92)	1.12 (1.00, 1.25)	0.04	0.01
Knee replacement	1.07 (0.85, 1.34)	0.91 (0.68, 1.21)	0.77 (0.50, 1.17)	0.92 (0.82, 1.03)	0.15	

^a^Adjusted for age, gender, body mass index, and country of birth using Cox's proportional hazard model.

^b^Test of homogeneity of trends for knee and hip joint replacement.

There was no statistical evidence that country of birth, gender, or educational level modified the associations between meat consumption and the risk of hip or knee replacement (P value for effect modification ranged from 0.11 to 0.92).

## Discussion

In this prospective cohort study, we found that a high level consumption of fresh red meat was associated with a decreased risk of hip, but not knee joint replacement for OA. Consumption of processed meat, chicken and fish had little association with the risk of either hip or knee joint replacement.

No previous study has examined the relationship between meat consumption and the risk of joint replacement, a proxy outcome measurement for severe OA. The data regarding the association between meat consumption and the risk of OA are sparse. In a community-based cross-sectional study, Kacar and colleagues found that the intake of meat/poultry and fish was not associated with the prevalence of symptomatic knee OA where the diagnosis of knee OA was made clinically or clinically and radiologically according to the American College of Rheumatology criteria [14]. In contrast, another cross-sectional study showed an independent and moderately increased prevalence of degenerative arthritis associated with greater meat, poultry, and fish consumption in both men and women [13]. However, this latter study used self-reported information on the prevalence of degenerative arthritis and rheumatism as outcome measure, including not only OA but also other forms of arthritis and rheumatism. Moreover, cross-sectional studies are subject to recall bias when trying to capture the dietary patterns which may contribute to the discrepancies. We used prospectively collected dietary data at two time points and followed up a large population for approximately 13 years to address the effect of meat consumption on the risk of joint replacement. In contrast to the findings from the two previous cross-sectional studies [13,14], we found that higher consumption of fresh red meat approximately 13 years prior to joint replacement was related to a decreased risk of hip, but not knee, joint replacement due to OA, whereas consumption of processed meat, chicken and fish was not associated with the risk. The association between fresh red meat consumption and hip replacement risk was further supported by the findings when self-reported meat consumption, recorded approximately 8 years prior to joint replacement, was examined. Similar effect (same direction and magnitude) of fresh red meat consumption was observed, although less significant due to reduced sample size.

The relationship between different food items and disease is sometimes difficult to interpret due to the inter-correlation between these items. Reported red meat consumption may be negatively correlated with reported consumption of white meat [23]. It has been recommended that red meat should be replaced by white meat, and the ratio of red meat to white meat consumption has been used as an indicator of compliance with this recommendation. Several studies on breast and colorectal cancer, including the Nurses Health Study [23] and the Health Professionals Follow-up Study [24], have placed great emphasis on this ratio. It has implications in the recommendation of consumptions of different food items and public health to examine the association between the ratio of red meat to white meat consumption and diseases. In our study we found consistent results for red meat consumption and the ratio of intake of fresh red meat to chicken and fish (Table 4).

Some methodological issues must be considered when interpreting the findings of this study. Residual confounders, dietary or non-dietary, may provide an alternative explanation for the observed associations. In this study, we have taken into account physical activity, smoking, and dietary intake of fruit, vegetable, vitamin C, vitamin E, beta-carotene, and polyunsaturated fatty acids as potential confounders. We have controlled for educational level and country of birth which may have captured some differences in terms of socioeconomic status in the analysis, since there is evidence that socioeconomic factors affect the utilization of joint replacement procedure [25,26]. However, it is possible that some residual confounding due to socioeconomic factors persists. Those of higher socioeconomic status are more likely to adhere to dietary recommendations regarding reduction in red meat intake. This would tend to bias the results towards an inverse relationship between meat consumption and the risk of joint replacement if residual confounding was the main explanation. Moreover, we would expect the same effect to be seen for knee and hip replacement which was not the case. In addition, the beneficial effect we observed was for fresh red meat but not for processed meat, suggesting that the effect is not simply due to residual confounding since consumption of these often follows similar socioeconomic patterns [27]. Multiple testing is another issue that needs to be considered, which may lead to the possibility of a spurious finding. When examining the association between meat consumption and the risk of joint replacement, we had 8 main analyses, 4 for the knee and 4 for the hip, for the association of fresh red meat, processed meat, chicken and fish. The ratio of fresh red meat to chicken and fish was a composite of the others and the finding was consistent. Thus using Bonferroni correction, a p value of 0.006 (0.05/8) would be considered significant. Our results were similar (Table 4). However, this correction does not take into account the dose-response relationship we observed, so needs to be considered with caution. Although there is the potential issue of multiple testing, this study is still a valid hypothesis generating study.

The strength of the study was the prospectively collected meat consumption data prior to joint replacement surgery. We also examined meat consumption at baseline and at first follow-up. At baseline, a FFQ was used which provided a validated accurate method for assessing meat consumption and determining energy intake. At first follow-up a simplified, limited questionnaire was administered to determine meat intake. Despite using the different methods, a similar effect was observed for fresh red meat consumption and the reduced risk of hip, but not knee replacement, providing further support for the beneficial effect of fresh red meat consumption on hip replacement risk and also suggesting that the observed effect was not dependent on the instrument used to determine meat intake. This is perhaps not surprising since we found a moderate correlation between baseline and follow up meat consumption in this healthy population, suggesting relatively stable meat consumption over the 4 years period, and supporting the notion that nutrient intake is relatively stable and tends to be more stable with increasing age [28,29].

Random error in measuring meat consumption at baseline and first follow-up may have occurred in this study, but is most likely to have attenuated the associations we observed. Because the FFQ did not measure portion sizes, the associations would be further attenuated if between-person differences in portion size contribute to between-person variability in amount consumed. Another potential limitation is the moderate reproducibility of the FFQ for the assessment of meat consumption, with kappa values ranged from 0.42 to 0.60 for the reliability study which assessed the FFQ twice at 12 months apart. However, the reliability is similar to others [30,31], and use of self-reported meat intake at MCCS baseline and first follow up provided similar results. Furthermore, this within-person error in reporting meat consumption is likely to be non-differential misclassification and thus may have diluted rather than identified a relationship. We did not have complete and reliable joint replacement data for the study participants prior to 2001. Although we excluded those MCCS participants who reported a joint replacement prior to 2001 at the second follow-up, this information may be unreliable and is only known for 68% of the original cohort. As a result, some misclassification of joint replacement status may have occurred. It is likely to have been non-differential in relation to the meat consumption measures and thus, may have attenuated any of the observed associations.

The mechanism for the relationship between fresh red meat consumption and the decreased risk of hip joint replacement at this stage is speculative. Bones are in a constant state of change through the process of remodeling which continues throughout life. Red meat is a rich source of protein and minerals, especially iron, zinc, selenium and magnesium, and a great source of vitamin B12 and long-chain omega-3 fatty acids. Iron, zinc, selenium and magnesium play an important role in bone metabolism and dietary intake of these minerals is positively associated with bone mass and strength [32]. Red meat is also a useful source of vitamin D which is essential for bone health. Higher animal protein consumption has been shown to be associated with increased bone mineral density and decreased bone loss [9,10]. There is increasing evidence that hip joint shape is modifiable and that subtle changes in hip joint shape are a risk factor for hip OA [33,34]. This is in contrast to knee OA where joint structure does not seem to be as important. It has been shown that certain deformities, such as the flattening of the femoral head, especially in conjunction with a sharper transition from the femoral head to the lower part of the neck are associated with increased risk of hip OA [34]. It may be that fresh red meat consumption reduces the risk of hip OA and subsequent hip replacement via increased bone strength and therefore less change in hip joint shape and less development of the types of hip deformities associated with hip OA. Whether this is the case will require further work.

## Conclusions

A high level consumption of fresh red meat was associated with a decreased risk of hip, but not knee, joint replacement for OA. One possible mechanism to explain these differential associations may be via an effect of meat intake on bone strength and hip shape. This should be taken as a hypothesis generating study, and further confirmatory studies are warranted.

## Abbreviations

OA: Osteoarthritis; MCCS: Melbourne Collaborative Cohort Study; FFQ: food frequency questionnaire; AOA NJRR: Australian Orthopaedic Association National Joint Replacement Registry; HR: hazard ratio; 95% CI: 95% confidence interval

## Competing interests

The authors declare that there are no competing interests.

## Authors' contributions

YW participated in the design of the study, performed the statistical analysis and the interpretation of data, and drafted the manuscript. JAS participated in the acquisition of data, helped to perform the statistical analysis, and reviewed the manuscript. AEW helped the interpretation of data, and reviewed the manuscript. DRE, GGG, and SG participated in the design of the study and the acquisition of data, and reviewed the manuscript. FMC participated in the design of the study, helped with the interpretation of data, and reviewed the manuscript. All authors read and approved the final manuscript.

## Pre-publication history

The pre-publication history for this paper can be accessed here:

http://www.biomedcentral.com/1471-2474/12/17/prepub
